# Spinal Afferent Innervation of the Colon and Rectum

**DOI:** 10.3389/fncel.2018.00467

**Published:** 2018-12-04

**Authors:** Stuart M. Brierley, Timothy J. Hibberd, Nick J. Spencer

**Affiliations:** ^1^Visceral Pain Research Group, Centre for Neuroscience, College of Medicine and Public Health, Flinders University, Bedford Park, SA, Australia; ^2^Centre for Nutrition and Gastrointestinal Diseases, Discipline of Medicine, South Australian Health and Medical Research Institute (SAHMRI), University of Adelaide, Adelaide, SA, Australia; ^3^Visceral Neurophysiology Laboratory, Centre for Neuroscience, College of Medicine and Public Health, Flinders University, Bedford Park, SA, Australia

**Keywords:** sensory nerve, spinal afferent, colon, sensory transduction, rectum, mouse, peripheral nervous system, dorsal root ganglia

## Abstract

Despite their seemingly elementary roles, the colon and rectum undertake a variety of key processes to ensure our overall wellbeing. Such processes are coordinated by the transmission of sensory signals from the periphery to the central nervous system, allowing communication from the gut to the brain via the “gut-brain axis”. These signals are transmitted from the peripheral terminals of extrinsic sensory nerve fibers, located within the wall of the colon or rectum, and via their axons within the spinal splanchnic and pelvic nerves to the spinal cord. Recent studies utilizing electrophysiological, anatomical and gene expression techniques indicate a surprisingly diverse set of distinct afferent subclasses, which innervate all layers of the colon and rectum. Combined these afferent sub-types allow the detection of luminal contents, low- and high-intensity stretch or contraction, in addition to the detection of inflammatory, immune, and microbial mediators. To add further complexity, the proportions of these afferents vary within splanchnic and pelvic pathways, whilst the density of the splanchnic and pelvic innervation also varies along the colon and rectum. In this review we traverse this complicated landscape to elucidate afferent function, structure, and nomenclature to provide insights into how the extrinsic sensory afferent innervation of the colon and rectum gives rise to physiological defecatory reflexes and sensations of discomfort, bloating, urgency, and pain.

## Introduction

Neural control of the colon and rectum is provided by distinct neuronal populations, whose cell bodies lie either within the gut wall (intrinsic; enteric neurons and viscerofugal neurons) (Furness, 2006, 2012) or outside it (extrinsic; sensory afferents or sympathetic neurons). Of most relevance to the direct generation of perceivable sensations, including pain, are the extrinsic sensory afferent neurons innervating the colon and rectum via the spinal nerves. Although, spinal afferents are generally associated with higher-threshold sensations such as discomfort, bloating, urgency, and pain, they are also equipped to convey information on physiological events (Harrington et al., 2018). These neurons also form the afferent limb of spinal and brainstem reflexes, enabling long range control of gastrointestinal motility and secretion through efferent pathways (Brierley et al., 2004; Brookes et al., 2013; Brierley and Linden, 2014).

Extrinsic sensory afferents that innervate the colon and rectum are subdivided based on the location of their soma. Splanchnic nerve cell bodies are located within the thoracolumbar dorsal root ganglia (DRG), whilst pelvic afferents have cell bodies within the lumbosacral DRG (Grundy and Brierley, 2018). Correspondingly, their central axons terminate within the dorsal horn of the thoracic, lumbar and sacral spinal cord, where they synapse with second order neurons (Sadeghi et al., 2018). To add further complexity the relative contributions of the pelvic and splanchnic innervations vary along the colon and rectum. The proximal colon is innervated by the thoracic and lumbar spinal cord via the lumbar splanchnic nerve (Harrington et al., 2018). In contrast, the distal colon has dual spinal innervation via both the lumbar splanchnic nerve and sacral pelvic nerves (Harrington et al., 2018). Based on nerve lesion studies (Kyloh et al., 2011) and retrograde transport studies (Grundy et al., 2018), the colorectum also receives predominant innervation from the lumbosacral spinal cord, via the sacral pelvic nerves, although there are contrasting reports depending on the species and retrograde tracer used (see: Christianson and Davis, 2010).

While not the focus of this review, we also note neuroanatomical evidence of a third, vagal source of afferent innervation that reaches distal colon in rats (Berthoud et al., 1990, 1997; Wang and Powley, 2007; Herrity et al., 2014). The colon represents the largest microbial reservoir in the body. Recent studies demonstrate that the effects of microbial composition on emotional behavior, and brain structure are vagus nerve-dependent, which has stimulated interest in this pathway (Bercik et al., 2011; Bravo et al., 2011). Electrophysiological recordings from this pathway have also recently been described (Buckley and O'Malley, 2018). Whether these pathways are prominent in other species remains to be demonstrated.

Besides the “pelvic” and “splanchnic” anatomical distinction of spinal afferents to the colorectum, numerous other classification schemes have also been applied to determine “sub-types” or “subclasses” of afferents (Brierley et al., 2004, 2008, 2009). Such studies have employed a variety of techniques including (i) electrophysiological recording methods alone (Brierley et al., 2004, 2008, 2009; Page et al., 2004, 2005, 2007; Hughes et al., 2009b; Osteen et al., 2016; Bellono et al., 2017; Castro et al., 2017) or (ii) electrophysiological recordings with subsequent anatomical analysis (Lynn et al., 2003; Spencer et al., 2008b; Zagorodnyuk et al., 2010; Lynn and Brookes, 2011; Humenick et al., 2015; Hibberd et al., 2016), or (iii) anatomical analysis without electrophysiological recordings (Spencer et al., 2014), or (iv) on the basis of gene expression alone (Hockley et al., 2018b). Therefore, a major task for afferent neurobiology is to integrate the various classification schemes applied to colonic afferents in order to identify basic afferent subtypes and ascertain a more unified classification scheme. Therefore, in this review, we aim to reconcile the complicated landscape of colonic sensory afferent structure, function, and nomenclature. This is important as sensory pathways innervating these organs are implicated in the aberrant sensation associated with common clinical gastrointestinal disorders. This includes the hypersensitivity of afferent pathways being linked with abdominal pain associated with organic diseases such as inflammatory bowel disease (Farthing and Lennard-Jones, 1978; Rao et al., 1987) of functional bowel disorders such as irritable bowel syndrome (Lembo et al., 1994). For a detailed review of the mechanisms involved see; (Brierley and Linden, 2014). Conversely, in humans, aging has been shown to be associated with impaired visceral sensory perception in response to mechanical stimulation of the rectum (Lagier et al., 1999), whilst diabetes induced fecal incontinence, is associated with impaired rectal sensation (Wald and Tunuguntla, 1984; Caruana et al., 1991).

## Distinct Classes of Sensory Afferents Innervate the Gastrointestinal Tract of Animals and Humans

The predominant classification scheme applied to murine colorectal afferents, has been the functional classifications that describe afferent capabilities to transduce and encode muscular distension, and/or mucosal deformation (Table 1; Brierley et al., 2004; Brierley, 2012). These classes include (1) muscular afferents (which respond in a wide dynamic range to circular stretch/distension from low thresholds), (2) low-threshold mucosal afferents (which are activated by distortion of the intestinal mucosa), (3) muscular/mucosal afferents (which respond to both circular stretch and mucosal distortion), and (4) vascular endings that wrap around blood vessels in the mesentery, and (5) serosa, or submucosal space, and respond to high-threshold stimuli (Brierley et al., 2004; Brookes et al., 2013; Brierley and Linden, 2014). A sixth afferent class, termed mechanically insensitive, or “silent afferents,” are activated by inflammatory or immune mediators (Table 1; Brierley et al., 2005a,b; Feng and Gebhart, 2011; Feng et al., 2012).

**Table 1:** Simple firing response profiles of the functional classes of afferents innervating the murine colon and rectum.

**Class**	**Stroke**	**Probe**	**Stretch**
Mucosal	Yes	Yes	No
Muscular mucosal	Yes	Yes	Yes–low threshold
Muscular	No	Yes	Yes–low threshold
Serosal	No	Yes	Yes–high threshold
Mesenteric	No	Yes	Yes–high threshold
Mechanically insensitive or “silent afferents”	No (in naïve conditions)	No (in naïve conditions)	No (in naïve conditions)

Studies of human ileum, appendix, colon (ascending, transverse, sigmoid, descending), and rectum have demonstrated the presence of afferents with function characteristics compatible with serosal (Jiang et al., 2011; Hockley et al., 2016; Yu et al., 2016; McGuire et al., 2017), mesenteric (Hockley et al., 2016; Yu et al., 2016; McGuire et al., 2017), muscular (Jiang et al., 2011; Yu et al., 2016; McGuire et al., 2017), muscular-mucosal (Jiang et al., 2011; McGuire et al., 2017), and mucosal afferents (Table 2; Yu et al., 2016). Studies by Peiris et al. (2011) and Ng et al. (2016) identified afferent firing in colon and rectum sensitive to focal probing but did not systematically test the effect of gut distension, or mucosal stroking, and therefore the mechanosensitive afferents identified in these studies could represent any of the mechanically-sensitive functional classes. Recruitment of “silent afferents” by inflammatory mediators has also been demonstrated in human colon (Peiris et al., 2011; Hockley et al., 2016; Yu et al., 2016; McGuire et al., 2017), including those which subsequently acquire mechanosensitivity (Ng et al., 2016). Serosal and muscular colorectal afferents are by far the most commonly reported afferent in human studies to date, with very few mucosal and muscular-mucosal afferents identified (Table 2). To date, no single study of human colorectal afferents has identified the full complement of functional classifications identifiable in rodents, and large numbers of unclassified afferents were reported by Yu et al. (2016). It is possible that technical limitations under which human tissue experimentation is done differentially affect survival among the functional afferent classes (aged and diseased subjects, with varying time in storage, and time between loss of tissue blood supply and immersion in oxygenated saline). Human afferent recordings performed to date have typically pooled data acquired from preparations derived from different locations along the entire colon, rectum, appendix, or ileum (Hockley et al., 2018a). Afferent sensitivity in human bowel, like that in rodents is also reduced with advancing age (Keating et al., 2016; Yu et al., 2016). Thus, it is currently unknown whether all functional classes of afferent are homogenously present along the large intestine. Overall, these studies suggest there are afferents with similarities in functional properties in both mouse and human, however the positive identification, and correlation with their underlying structures in both species is also required.

**Table 2:** Functional classes of afferents innervating the human intestine and rectum.

	**Region Number of preparations (number of successful recordings)**							**Stimuli applied**			**Functional classification Number of units**							
**Study**	**Ileum**	**Appendix**	**Ascending**	**Transverse**	**Descending**	**Sigmoid**	**Rectum**	**Probe**	**Stretch**	**Stroke**	**Units**	**Mucosal**	**Muscular Mucosal**	**Muscular**	**Serosal**	**Mesenteric**	**Silent**	**Unclassified**
Hockley et al., 2016	6	7	–	–	15	–	–	Yes	Yes	Yes	ND	0	0	0	3	1	α	ND
Jiang et al., 2011	–	–	27 (4)	–	–	–	–	Yes	Yes	Yes	5	0	1	2	2	0	0	0
McGuire et al., 2017	(4)		(1)	(1)	(1)	(23)	(5)	Yes	Yes	Yes	46	0	1	18	23	2	2	0
Ng et al., 2016	–	–	–	5	5	–	8	Yes	No	No	ND	N/A	19^*^			N/A	3^*^	ND
Peiris et al., 2011	–	18 (9)	9 (4)	–	–	–	–	Yes	No	No	ND	N/A	2^*^			0	β	ND
Yu et al., 2016	10 (6)	–	4 (2)	–	5 (5)	12 (5)	2 (1)	Yes	Yes	Yes	34	1	0	2	5	2	2	24

* *Number of focal hotspots, number of units undefined. α silent afferents represented the majority of recorded units, number undefined. β silent afferents identified, number undefined. N/A, not applicable; ND, no data*.

In the mouse, all sensory afferent classes innervating the colon and rectum have nerve conduction velocities within the C-fiber range, whilst the vast majority of afferents are also peptidergic (Jones et al., 2005; Brierley et al., 2008, 2009). As discussed below, the peripheral endings of many afferents display specialized anatomical structures, rather than the “free nerve endings” that they were traditionally thought to possess (Brookes et al., 2013). Afferents that do have free nerve endings appear to correspond to the putative nociceptive vascular/serosal/mesenteric class, whose simple endings terminate adjacent the gut and mesenteric vasculature (Brunsden et al., 2007; Song et al., 2009; Humenick et al., 2015). The known specialized endings include the long studied intraganglionic laminar endings of vagal (Lawerentjew, 1929; Nonidez, 1946; Rodrigo et al., 1975; Berthoud et al., 1995), and spinal origin (Lynn et al., 2003; Olsson et al., 2004) that correspond with low-threshold tension receptors. There are also specialized “intramuscular arrays” identified in rat and guinea pig vagal (Berthoud and Powley, 1992; Kressel et al., 1996; Fox et al., 2000; Wang and Powley, 2000) and spinal nerves (Lynn et al., 2003; Olsson et al., 2004), as well as vagal “web-like” endings in rat (Powley et al., 2012, 2013). Vagal intramuscular arrays and web-like endings have not been correlated with functional studies, however spinal intramuscular arrays have been correlated with muscular afferents of the guinea pig internal anal sphincter (Lynn and Brookes, 2011). Recent advances in selective neuroanatomical tracing of visceral afferents in mice have further identified complex arrays of endings whose functional properties remain to be identified (Brookes et al., 2013; Spencer et al., 2014, 2016). This is important as this diversity in ending structure is a contributing factor to the vastly different sensory functions of these afferent classes. Another contributing factor to this diverse function is the different cohorts of ion channels and receptors expressed by these afferents (**Figure 2A**; Brierley et al., 2004; Erickson et al., 2018; Sadeghi et al., 2018), as discussed below.

Functional classifications currently applied to murine afferents are built upon a large body of earlier work conducted in other species. Initial electrophysiological recordings from visceral afferent nerves of cats, rabbits, and frogs were reported in the 1930's (Adrian et al., 1932; Tower, 1933; Gammon and Bronk, 1935). Development of single fiber afferent recordings (Paintal, 1953), enabled a series of studies by Paintal and Iggo utilizing stomach and small intestine. They described the low threshold vagal muscular afferents in cat, sheep, and goat (Paintal, 1953, 1954a,b; Iggo, 1955, 1956, 1957b) and mucosal receptors in cat (Iggo, 1957a; Paintal, 1957). Muscoal afferent endings, presumed to be located in the mucosa as they were ablated by its removal, while muscular afferents persisted and were inferred to be located in the outer musculature (Iggo, 1957a). Afferents with combined properties of muscular and mucosal receptors were later described by Harding and Leek in sheep (Harding and Leek, 1972). In rat upper gastrointestinal tract, muscular, and mucosal afferents were characterized in a series of reports by Clarke and Davison (1974, 1975, 1976, 1978). Major contributions followed by Andrews, Scratcherd, Grundy, Blackshaw, and their collaborators who revealed in ferret and rats the detailed stimulus-response properties of mechanosensitive afferents (Andrews et al., 1980; Blackshaw et al., 1987b; Lynn and Blackshaw, 1999), chemosensitive afferents (Blackshaw and Grundy, 1990, 1993; Grundy et al., 1995; Richards et al., 1996; Eastwood et al., 1998), and their roles in autonomic reflexes (Grundy et al., 1981; Blackshaw et al., 1987a; Blackshaw and Grundy, 1988). These topics have been extensively reviewed (Andrews, 1986; Grundy and Scratcherd, 1989; Blackshaw et al., 2007; Grundy and Schemann, 2007).

Visceral pain and the search for visceral nociceptors has long been a topic of interest (Ness and Gebhart, 1990; Cervero, 1994) Early studies demonstrated major differences with somatic pain. Strong gut distension, chemical irritants, and heat could evoke visceral pain (Ness and Gebhart, 1990). However, other stimuli such as cutting, pinching, burning, and piercing—all strongly pain-evoking applied to skin or muscle—were not painful when applied to viscera in animals and conscious humans (von Haller, 1755; Lennander, 1902; Carlson and Braafladt, 1915). While this may be in part due to the experimental conditions (Ness and Gebhart, 1990), the mesentery and its vasculature, by contrast, was commonly reported as exquisitely sensitive to mechanical deformation by traction or by ischaemia, causing intense, nauseating pain (Lennander, 1902; Gray, 1922; Morley, 1931). The sensitivity of visceral afferents to strain of the mesenteric vasculature was noted among the earliest visceral afferent recordings (Tower, 1933; Gernandt and Zotterman, 1946). Detailed electrophysiological studies by Bessou and Perl (1966), Floyd and Morrison (1974), Floyd et al. (1976), and Morrison (1973) localized the vasculature branching points in mesentery and locations on the gastrointestal serosa as focal mechanotransduction sites of c fiber afferents. Furthermore, Song et al. showed mechanotransduction sites of these afferents correlated with free paravascular nerve endings, whether in the mesentery (“mesenteric” afferents) or gut (“serosal” afferents) (Song et al., 2009).

## Muscular Afferents

**Figure 1:**
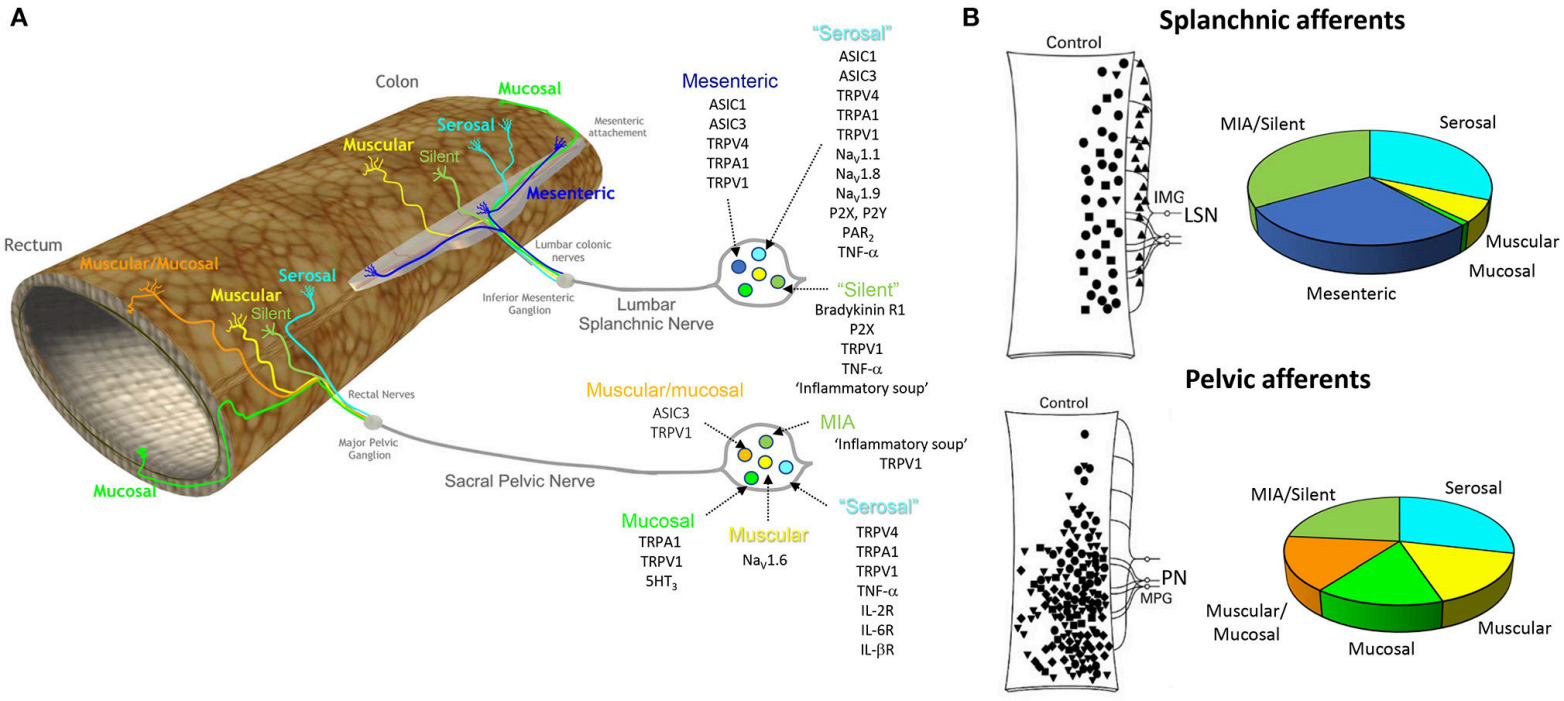
Different classes of afferent innervating the colon and rectum and the ion channels and receptors contributing to their function. **(A)** The colon and rectum are innervated by two distinct spinal pathways, the lumbar splanchnic and sacral pelvic nerves. The cell bodies of these splanchnic and pelvic afferents are located within the thoracolumbar (T10-L1) and lumbosacral (L6-S1) DRG, respectively. Six broad classes of afferents exist. (1) mesenteric (splanchnic only), (2) muscular/mucosal (pelvic only), (3) serosal (splanchnic and pelvic pathway), (4) muscular (splanchnic and pelvic pathway), (5) mucosal (splanchnic and pelvic pathway) (Brierley et al., 2004), and (6) mechanically insensitive ‘silent’ afferents (splanchnic and pelvic pathway), which lack mechanosensitivity in naïve conditions but are recruited by chemical stimuli (Brierley et al., 2005a; Feng and Gebhart, 2011). A key list of excitatory ion channels and receptors that contribute to afferent function are listed for each subclass (Brierley, 2010). **(B)** Distribution of the receptive fields of afferent endings throughout the distal colon and rectum from Hughes et al. (2009b) with permission, with the percentages of the respective afferent classes recorded within the splanchnic and pelvic nerves (Brierley et al., 2004; Hughes et al., 2009b; Feng and Gebhart, 2011). ASIC, Acid sensing ion channel; TRP, transient receptor potential channel (TRP); vanilloid 1 (TRPV1), vanilloid 4 (TRPV4), ankyrin 1 (TRPA1), voltage-gated sodium channel: Nav, MIA, mechanically insensitive afferents; TNF-a, tumor necrosis α, interleukin receptor (IL-R); P2X, ligand-gated ion channel; P2Y, G-protein-coupled purinoceptor; 5-HT3, 5-Hydroxytryptamine receptor 3; PAR2, protease activated receptor 2. Bradykinin R1, Bradykinin receptor 1.

Muscular afferents in the colon and rectum, whether recorded using *ex vivo* rodent preparations or *in vivo* in the splanchnic or pelvic nerves of cats and rats (Blumberg et al., 1983; Jänig and Koltzenburg, 1991; Sengupta and Gebhart, 1994a) display similar response profiles. In the colon and rectum, muscular afferents respond to low distension pressures (< 20 mm Hg); (Malin et al., 2009) or low-intensity stretch stimuli (< 3 g) within the physiological range (Table 1; Figure 1A; Brierley et al., 2004; Hughes et al., 2009b). Muscular afferents are more prevalent within the pelvic innervation where they represent 21% of all mechanosensitive afferents, and 17% of all afferents (Brierley et al., 2004; Feng and Gebhart, 2011). Muscular afferents are relatively rare in the splanchnic pathway representing 10% of all mechanosensitive afferents and 6% of all afferents (see Figure 1; Brierley et al., 2004; Hughes et al., 2009b; Feng and Gebhart, 2011). Pelvic muscular afferents are found in both the distal colon and rectum and adapt more slowly to maintained distension compared to splanchnic muscular afferents, which are only found in the distal colon (Brierley et al., 2004; Hughes et al., 2009b; Feng and Gebhart, 2011). The anatomical transduction sites of rectal muscular afferents have been identified in the guinea pig as flattened branching endings in the myenteric ganglia called rectal intraganglionic laminar endings (or rIGLEs). Morphologically they appear similar to IGLEs innervating the stomach via the vagus nerve, but are smaller in size, less complex in structure, and are non-peptidergic (Brookes et al., 2013, 2016; Spencer et al., 2014, 2016). Muscular afferents are activated by contraction of either the circular or longitudinal muscle of the colon and rectum. Noteably, murine rectal muscular afferents have significantly greater stretch-responses than colonic muscular afferents suggesting that the encoding of mechanosensory information differs between colonic and rectal stretch-sensitive pelvic afferents (Feng et al., 2010). Therefore, muscular afferents likely respond to physiological levels of distension caused by the passage of fecal matter in the distal colon and particularly the rectum, thereby contributing to defecatory reflex pathways (Harrington et al., 2018). Indeed, low amplitude (non-painful) distensions of human rectum is well known to evoke a sensation of fullness followed by an urge to defecate (Hurst, 1911; Boring, 1915; Kwan et al., 2002; De Ocampo et al., 2007; Gundling et al., 2010).

The voltage-gated sodium channel Na_V_1.6 has been demonstrated to play a key role in the function of pelvic muscular afferents (Figure 1; Harrington et al., 2018). Na_V_1.6 protein is expressed by the cell soma of colon-innervating sensory neurons, whilst Na_V_1.6 is expressed by afferent nerve endings within the distal colon and rectum of mice (Feng et al., 2015). Using pharmacological blockers of Na_V_1.6 reduces the number of action potentials fired in response to stretch of the distal colon and rectum (Feng et al., 2015).

## Mucosal Afferents

Mucosal afferents in the colon and rectum are generally silent and do not fire action potentials at rest. However, they generate a brief burst of action potentials in response to very light stroking or compression of the mucosa in the colon or rectum (Brierley et al., 2004). The magnitude of this response increases proportionally with increasing stimulus strengths applied to their receptive fields (Brierley et al., 2004). Mucosal afferents are not sensitive to distension or contraction (Table 1; Brierley et al., 2004). As mucosal afferents display a remarkable sensitivity to low-threshold stimuli applied to the mucosa, they likely play a crucial mechanosensory role in detecting the particle size of luminal contents (Harrington et al., 2018).

Within the colon, mucosal afferents are rare representing only 4% of mechanosensitive afferents (Brierley et al., 2004) and 1% (Feng and Gebhart, 2011) of the total afferents recorded from the splanchnic nerves of the mouse (Figure 1). In contrast, mucosal afferents within the distal colon and rectum are more frequently observed in the pelvic innervation. Overall, mucosal afferents represent 25% of mechanosensitive afferents and ~15% of all afferents recorded within the pelvic nerve (Brierley et al., 2004; Hughes et al., 2009a; Feng and Gebhart, 2011). The increased abundance of mucosal afferents in the distal colon and rectum suggests an important role in transmitting information about stool consistency as material passes from the proximal to distal colon and into the rectum (Harrington et al., 2018). Therefore, pelvic mucosal afferents likely contribute to the control of defecation, via conscious perception of stool passage in the rectum and anal canal (Jänig and Koltzenburg, 1991; Sengupta and Gebhart, 1994a). In mice, the anatomy of pelvic mucosal afferents has recently been identified, with fibers consisting of single or branched fine varicose axons that ramify within the mucosa. These studies revealed that 11% of all the pelvic afferent endings project into the mucosa. Of these mucosal endings, 90% were found to express calcitonin gene-related peptide (CGRP), and are therefore peptidergic (Spencer et al., 2014).

In terms of key channels contributing to mucosal afferent function, TRPA1 (transient receptor potential ankyrin 1) is located in mucosal nerve fibers (Brierley et al., 2009), whilst TRPA1 agonists (cinnamaldehyde or mustard oil) evoke mechanical sensitization of pelvic mucosal afferents (Brierley et al., 2009). Correspondingly, mucosal afferents from TRPA1^−/−^ mice display deficits in action potential firing to mucosal stimulation (Brierley et al., 2009) (Figure 1). Compatible with a role for mucosal afferents in defecation control, mustard solution, which contain TRPA1 agonists, evoked the urge to defecate upon application to the human rectum (Boring, 1915) whilst intracolonic allyl isothiocyanate (TRPA1 agonist) evoked colonic motility and defecation in dogs (Someya et al., 2015).

More recently, it has been demonstrated that mucosal afferents can communicate directly with enterochromaffin cells, which are key cells within the epithelial lining of the colon and rectum. Enterochromaffin cells express a wide variety of channels and receptors, including the odorant receptor Olfr588 and the α2A adrendoreceptor (Adrα2A) (Bellono et al., 2017). Activation of these receptors by the microbial metabolite isovalerate, or by norepinephrine, respectively, results in 5-HT (5-hydroxytryptamine, serotonin) release from enterochromaffin cells. This 5-HT then activates 5-HT_3_ receptors expressed on mucosal afferents to induce mechanical hypersensitivity (Figure 1; Bellono et al., 2017). Accordingly, mucosal afferents play a key role in detecting both the mechanical and chemical environment within the distal colon and rectum.

## Muscular/Mucosal Afferents

As discussed above, distinct classes of mucosal and muscular afferents have been clearly identified. However, there also exists a separate distinct class of afferent that display the properties of both muscular and mucosal afferents (Brierley et al., 2004). That is, they respond to both low-threshold distension or contraction of the colon and rectum in addition to light mucosal distortion of the mucosa (Table 1; Harrington et al., 2018). In the mouse, these spinal muscular/mucosal afferents occur only within the pelvic innervation of the distal colon and rectum (Figure 1). Muscular/mucosal afferents are not present within the splanchnic innervation, but represent ~25% of mechanosensitive and 17% of the total pelvic innervation (Brierley et al., 2004; Hughes et al., 2009b; Feng and Gebhart, 2011). Together with their low-distension thresholds, this suggests they may also contribute to spinal defecatory circuits and conscious sensation (Harrington et al., 2018). Recent anatomical anterograde tracing studies have identified a remarkably complex array of different pelvic afferent ending morphologies across the various layers of the colorectum. Among those are endings within the Crypts of Lieburkuhn and the submucosal ganglia, which may represent the anatomical correlate of muscular/mucosal afferents (Spencer et al., 2014).

The acid sensing ion channel ASIC3 and the transient receptor potential channel TRPV1 play key roles in the function of pelvic muscular/mucosal afferents (Figure 1). Muscular/mucosal afferents from ASIC3^−/−^ and TRPV1^−/−^ mice have significantly reduced mechanical sensitivity, compared with those in wild-type mice. *In vivo* these decreases in mechanosensory function correlate with reduced pain responses to colorectal distension (Jones et al., 2005). Intra-colonic zymosan induces elevated pain response to colorectal distension in mice. However, this sensitizing action is lost in ASIC3^−/−^ mice (Jones et al., 2007), suggesting ASIC3 also contributes to peripheral sensitization in the colon and rectum (Brierley, 2010).

## Vascular, Serosal and Mesenteric Afferent Endings

Afferents with receptive fields on the mesenteric attachment and serosa were originally described in recordings from splanchnic nerves (Bessou and Perl, 1966; Morrison, 1973; Blumberg et al., 1983). Mesenteric afferents fire action potentials to focal compression or stretch of the mesentery (Table 1; Morrison, 1973; Blumberg et al., 1983). Depending on the species recorded from, single mesenteric afferent neurons can have up to seven punctate receptive fields (Morrison, 1973; Blumberg et al., 1983; Jänig and Koltzenburg, 1991; Sengupta and Gebhart, 1994a,b; Lynn and Blackshaw, 1999). Mesenteric afferents often show rapidly adapting distension-evoked firing responses and have distension-response thresholds in the noxious range (Blumberg et al., 1983; Brierley et al., 2004, 2008, 2009; Hughes et al., 2009b). Therefore, mesenteric afferents likely contribute to signaling mechanically induced pain and display mechanical hypersensitivity in disease states (Hughes et al., 2009b). Mesenteric afferents are specific to the splanchnic innervation, where they represent ~50% of all mechanosensitive afferents and ~30% of all splanchnic afferents innervating the colon (Brierley et al., 2004; Hughes et al., 2009b; Feng and Gebhart, 2011) Mesenteric afferents have not been identified from the pelvic innervation (Table 1; Brierley et al., 2004, 2005a).

Earlier studies of serosal afferents described them as being “indistinguishable” from mesenteric afferents, other than by the locations of their receptive fields (Morrison, 1973). However, recent studies have shown they are distinct (Brierley et al., 2004). These afferents were speculated to terminate in the serosa (hence “serosal”). However, the term is likely a misnomer, as their punctate transduction sites have been correlated with paravascular nerves both in mesentery and in the submucosa (Brookes et al., 2013, 2016; Spencer et al., 2014, 2016). Similarly, despite an abundance of high-threshold “serosal” afferents in pelvic pathways (Brierley et al., 2004), selective tracing of pelvic afferents did not reveal nerve terminals in the serosal layer (Spencer et al., 2014).

Collectively, this class of afferents has been referred to as “serosal”, “nociceptors”, “high-threshold,” and “vascular” (Brierley et al., 2004; Song et al., 2009; Brookes et al., 2013; Castro et al., 2013, 2017; Harrington et al., 2018). Generally, these afferents do not respond to low-threshold mechanical stimuli and respond to noxious intensities of distension (>40 mm Hg; Brierley et al., 2008) or stretch (>9 g; Hughes et al., 2009b; see Table 1). Such properties match the original *in vivo* reports of colonic afferents with high-thresholds to distension (Sengupta et al., 1990; Sengupta and Gebhart, 1994a,b). Based on their physiological response profiles these “vascular/serosal” and “mesenteric afferents” display the properties of high-threshold mechanonociceptors (Harrington et al., 2018). Unlike mesenteric afferents, serosal afferents are common to both the splanchnic and pelvic pathways, representing ~33% of all the mechanosensitive afferents and ~29% of all afferents in these respective pathways (Table 1).

Vascular/serosal high-threshold afferents not only respond to high-threshold mechanical stimuli, but also respond to a wide variety of inflammatory and immune mediators, including bradykinin, tumor necrosis factor alpha (TNF-α), interleukin (IL)-2, IL-6, IL-1β (Brierley et al., 2005b; Hughes et al., 2013; Campaniello et al., 2016) and activators for purinoreceptor subtypes P2X, P2Y, Protease activated receptor 2 (PAR_2_), and TRPV1 (Brierley et al., 2005a; Sipe et al., 2008; Hockley et al., 2016). Numerous ion channels including voltage-gated sodium channels (Na_V_1.1, Na_V_1.8, Na_V_1.9), ASIC3, TRPV4, TRPA1, TRPV1 are all integral to high-threshold afferent function and also contribute to afferent sensitization (Page et al., 2005, 2007; Brierley et al., 2008, 2009; Hockley et al., 2016; Osteen et al., 2016; Inserra et al., 2017; Salvatierra et al., 2018). These afferents also display mechanical hypersensitivity in inflammatory and chronic visceral hypersensitivity states (Table 1; Brierley et al., 2009; Hughes et al., 2009a, 2014; Castro et al., 2013, 2017; Brierley and Linden, 2014; de Araujo et al., 2014; Osteen et al., 2016).

## Mechanically Insensitive “Silent” Afferents

Numerous studies have reported the existence of colonic afferents that are initially mechanically-insensitive, but subsequently respond to mechanical stimuli following exposure to chemicals and inflammatory mediators (Table 1; Brierley et al., 2005a,b; Feng and Gebhart, 2011). In naïve preparations from mice ~33% of all splanchnic and ~23% of pelvic afferents in the distal colon and rectum are mechanically insensitive (Figure 1; Feng and Gebhart, 2011). Treatment of mice with zymosan leads to a decrease in the proportion of “silent” afferents at both short and long-term time points post-zymosan treatment (Feng and Gebhart, 2011). Interestingly, there is a corresponding increase in the proportion of mechanically sensitive high-threshold vascular/serosal afferents at the same time points, suggesting the mechanically insensitive afferent become sensitized and develop the properties of vascular/serosal afferents (Harrington et al., 2018). The increase in the proportion of vascular/serosal afferents following insult may increase the afferent barrage from the periphery to the spinal cord in response to distension and contraction, resulting in persistent pain states.

Overall, there is evidence for multiple types of silent afferents, this includes mechanically insensitive afferents that respond to chemical stimuli, but do not subsequently become mechanosensitive (Brierley et al., 2005a). Secondly, there are silent afferents that are not chemically activated, but are mechanically sensitized (Feng and Gebhart, 2011) and thirdly, silent afferents that are chemically activated and mechanically sensitized (Figure 1; Feng and Gebhart, 2011). Collectively these observations suggest that mechanosensory proteins may exist in vascular/serosal afferent endings that are quiet in naïve situations, but can develop functionality following inflammatory insult. This process would therefore allow the afferent to respond to mechanical stimuli after the insult (Harrington et al., 2018). Recently it was demonstrated that “silent” nociceptors in the skin are characterized by the expression of the nicotinic acetylcholine receptor subunit alpha-3 (CHRNA3) (Prato et al., 2017). In these neurons, nerve growth factor induces Piezo2-dependent mechanosensitivity. Retrograde tracing studies show that CHRNA3 (+) neurons innervating deep somatic tissues and visceral organs represent ~50% of all peptidergic nociceptive afferents (Prato et al., 2017).

## Profiling Murine Gastrointestinal Spinal Afferents Using *ex vivo* DRG-gut Preparations

Little is known of how neuroanatomical structures directly relate to the functional classifications described above (Spencer et al., 2014). The combined application of physiological-mapping of sensory transduction sites with neuroanatomical tracing of afferent terminals in the guinea pig has been critical for identifying the types of mechanosensory endings that exist in that species (Zagorodnyuk and Brookes, 2000; Zagorodnyuk et al., 2001; Lynn et al., 2003; Song et al., 2009; Lynn and Brookes, 2011). As discussed above at least 5 distinct classes of mechanosensitive gastrointestinal afferent endings may occur (Brookes et al., 2013). These include the low threshold, tension-sensitive intraganglionic laminar endings (Zagorodnyuk and Brookes, 2000; Zagorodnyuk et al., 2001; Lynn et al., 2003), medium/high-threshold vascular afferents (Song et al., 2009; Humenick et al., 2015), low-threshold intramuscular arrays (Lynn and Brookes, 2011), low-threshold muscular-mucosal afferents (Brookes et al., 2013), and mucosal afferents (Brookes et al., 2013). The latter two classes currently lack direct correlations between sensory transduction sites and their neuroanatomical structures. This is also the case for mechanically insensitive afferents from the splanchnic and pelvic nerves.

**Figure 2:**
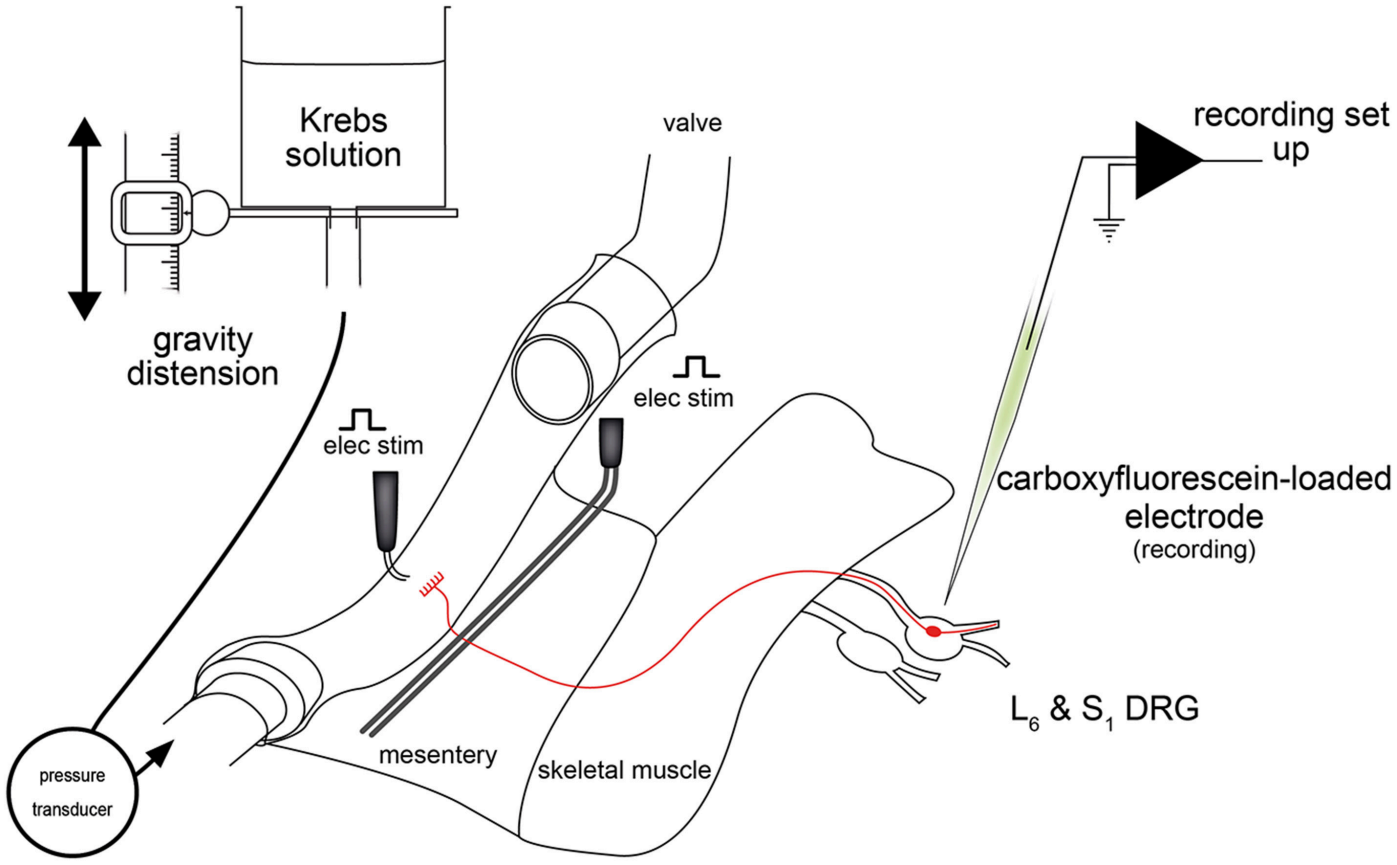
Schematic diagram of the *ex vivo* DRG-gut preparation used for recording lumbosacral colorectal afferent nerve cell bodies (Hibberd et al., 2016). Nerve cell bodies were randomly impaled and assessed for antidromic action potentials evoked by electrical stimulation of the mesentery. Neurons with positive responses were further characterized mechanically, electrophysiologically and neurochemically. Figure modified from Hibberd et al. (2016).

The extensive availability of gene technologies makes highly desirable a similarly detailed understanding of the function of murine visceral afferents and the morphological structures that underlie them. It is reasonable to expect that similar afferents occur in mouse as has been reported in the guinea pig, but direct evidence is elusive. A major obstacle to obtaining such evidence in mouse is a high density of afferents and other neurons supplying the gut. This makes the difficult task of identifying single afferent nerve endings in combined physiological-morphological studies. Nevertheless, preliminary studies combining electrophysiological recordings of single afferent neurons with neuroanatomical tracing have been conducted (Spencer et al., 2008a,b). More recently, *ex vivo* DRG-gut preparations have been employed by Malin et al. (2009) and Hibberd et al. (2016) (Figure 2). This alternative approach enables single cell electrophysiological characterization to be combined with studies of nerve cell body morphology and neurochemistry.

**Figure 3:**
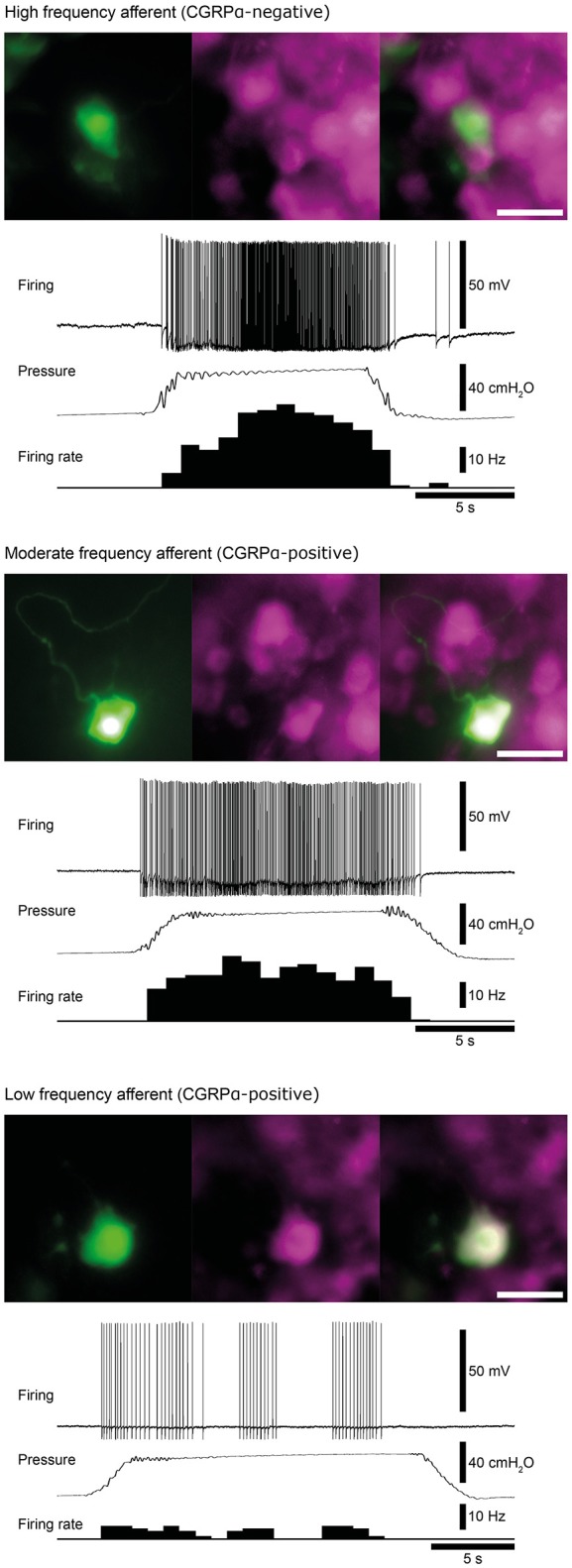
Examples of high, moderate and low frequency afferents. Matched micrographs show the nerve cell body morphology revealed by carboxyfluorescein (green) and CGRPα content (magenta). The firing response of each cell to a 40 cmH_2_O distension of the colorectum are shown. Calibration, 50 μm. Figure modified from Hibberd et al. (2016).

*Ex vivo* DRG-gut preparations comprise individual or multiple DRG, spinal nerve pathways, and a segment of gut. These preparations are sharp dissected free from the body and setup for intracellular electrophysiological recordings (Figure 2). Thus, electrophysiological recordings are made from the nerve cell bodies of gastrointestinal spinal afferent neurons with their connections to the gut intact (Figure 3). Similar preparations, were developed and used extensively for studies of cutaneous afferents (Ritter et al., 2000; Woodbury et al., 2001) before adaptation to gut studies (Malin et al., 2009). Since a minority of all spinal afferent neurons make projections to the viscera (Cervero et al., 1984; Robinson et al., 2004), a robust method of discriminating gut-innervating afferents is required in DRG-gut preparations before the application of mechanical or chemical stimuli. This may be done by recording action potential firing responses to electrical stimulation of extrinsic nerve pathways during random impalements of DRG nerve cell bodies.

Conventional extracellular electrophysiological recordings of spinal afferent axons are relatively high throughput, robust to mechanical disturbances and can be combined with rapid neuronal tracing of endings after recording (Zagorodnyuk and Brookes, 2000). On the other hand, intracellular recordings afford an opportunity to observe electrophysiological characteristics in greater detail. They also provide single cell recordings without the need to discriminate firing among multiple cells, and readily allow nerve cell body morphological, and neurochemical characteristics to be assessed, and related to mechanosensory, and chemosensory characteristics. It also possible to trace central spinal projections of DRG neurons, enabling correlation of afferent functional properties with their central neuroanatomy. This possibility remains to be exploited in *ex-vivo* DRG-gut preparations, but has been performed extensively in cutaneous afferents (Woodbury et al., 2001, 2004, 2008; Woodbury and Koerber, 2003, 2007; Albers et al., 2006).

Murine spinal afferents that innervate the colorectum have been recorded in two studies utilizing *ex vivo* DRG-gut preparations. These studies recorded pelvic colorectal afferents with nerve cell bodies in the L6 (Malin et al., 2009; Hibberd et al., 2016), and S1 DRG (Hibberd et al., 2016). Select characteristics of distension-sensitive afferents recorded by Malin et al. (2009) and Hibberd et al. (2016) are compared in Table 3 and described here.

**Table 3:** Properties of murine colorectal afferents recorded in L6 and S1 DRG with intact connections to the gut.

		**Hibberd et al., 2016**	**Malin et al., 2009**
High frequency	% of distension-sensitive cells Distension threshold (cm H_2_O) MFR at 40 cm H_2_O (Hz) Action potential amplitude (mV) Action potential half-peak duration (ms) Neurochemistry Nerve cell body size (μm^2^)	27 < 10 12.9 ± 1.1 57 ± 1.1 1.8 ± 0.2 CGRPα-neg 635 ± 62	29 2.6 ± 0.4 11 ± 1.5 59 ± 0.5 1.8 ± 0.1 83% TRPV1-neg, 87% GFRα3-neg –
Moderate frequency	% of distension-sensitive cells Distension threshold (cm H_2_O) MFR at 40 cm H_2_O (Hz) Action potential amplitude (mV) Action potential half-peak duration (ms) Neurochemistry Nerve cell body size (μm^2^)	41% < 10 7.3 ± 0.4 63 ± 1.9 ± 0.03 CGRPα-pos 796 ± 79	
Low frequency	% of distension-sensitive cells Distension threshold (cm H_2_O) MFR at 40 cm H_2_O (Hz) Action potential amplitude (mV) Action potential half-peak duration (ms) Neurochemistry Nerve cell body size (μm^2^)	32 < 10 1.7 ± 0.1 63 ± 2.2 1.7 ± 0.7 CGRPα-pos 889 ± 90	71 17.5 ± 1.1 1.4 ± 0.4 62 ± 1.6 2.3 ± 0.1 86% TRPV1-pos, 41% GFRα3-pos -

*MFR, mean firing rate; CGRPα, calcitonin gene-related peptide α; GFRα3, glial cell line-derived neurotrophic factor (GDNF) family receptor α 3; TRPV1, transient receptor potential vanilloid 1; DRG, dorsal root ganglion*.

**Figure 4:**
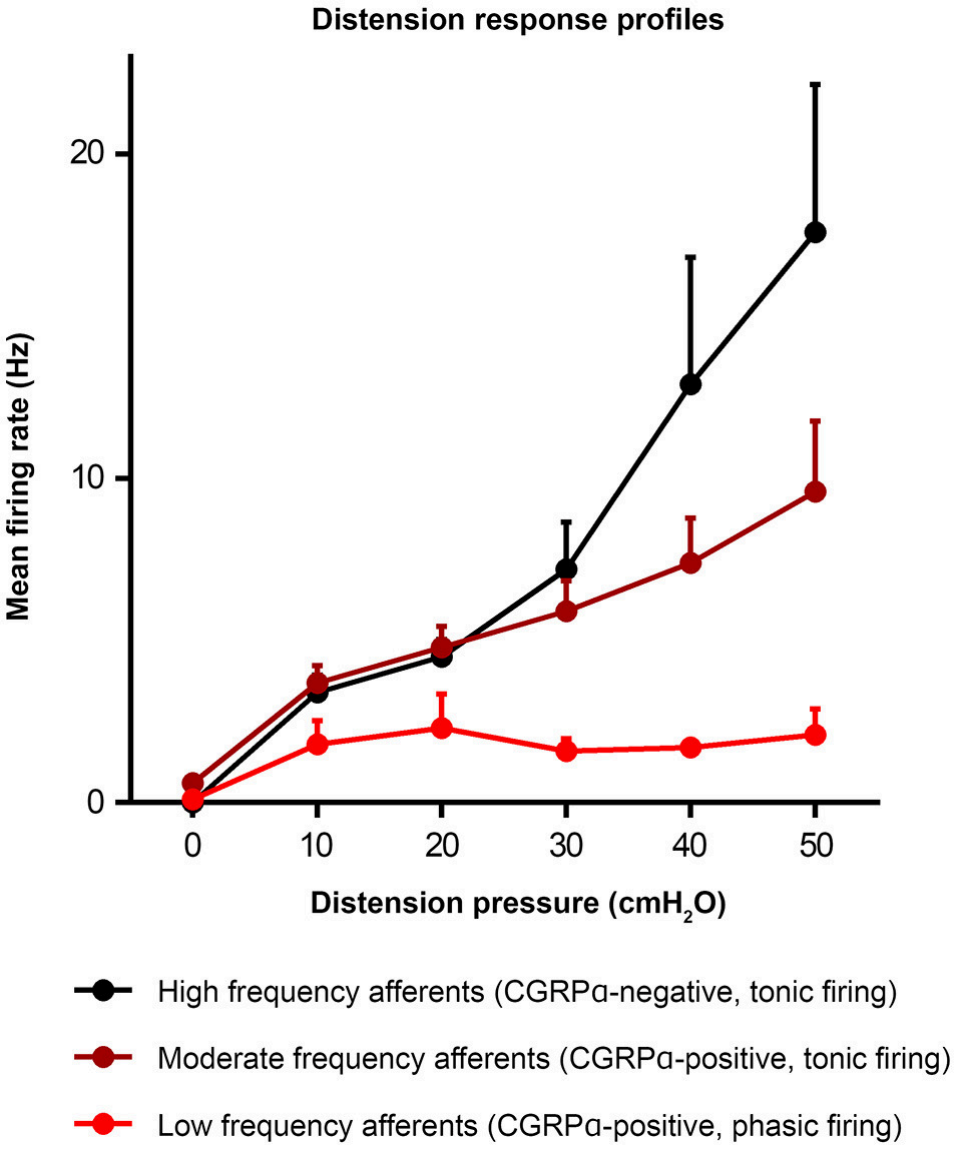
Distension response profiles of high, moderate, and low frequency afferents (Hibberd et al., 2016). Graph modified from original in Hibberd et al. (2016).

Similar populations of distension-sensitive colorectal afferent neurons recorded from L6 DRG were referred to as “high frequency” cells in both Malin et al. (2009), and Hibberd et al. (2016) (Figures 3, 4). High frequency afferents in both studies comprised similar proportions of all colorectal afferents in both studies (27–29%), and had the steepest distension-response firing profiles with wide dynamic ranges and low distension-response thresholds. Additionally, they fired with similar average frequencies at 40 cm H_2_O and had similar action potential half-peak durations (Table 3). The high degree of CGRP and TRPV1 colocalization in the mouse colorectum suggests that afferents lacking TRPV1 are also likely to lack CGRP (Sharrad et al., 2015). Interestingly, the majority of high frequency afferents recorded by Malin et al. (2009) lacked TRPV1- and GFRα3-immunoreactive content, while all high-frequency afferents recorded by Hibberd et al. lacked CGRPα Hibberd et al. (2016).

Distension-sensitive afferents with low thresholds and wide dynamic ranges fit into the functional classifications of “muscular” and “muscular/mucosal” (Brierley et al., 2004). Whilst it remains to be definitively demonstrated which of these classes the high frequency afferents belong to, there is evidence that muscular/mucosal afferents can be separated into low and high frequency firing sub-populations (Brierley et al., 2004). Interestingly, intraganglionic laminar endings are one of few colorectal afferent nerve terminals in mouse colorectum that lack CGRP and have nerve cell bodies in the L6 ganglia (Spencer et al., 2014). In guinea pig, rectal IGLEs are low threshold distension-sensitive mechanoreceptors that lack capsaicin-sensitivity (Lynn et al., 2003). It is possible that high frequency afferents recorded by Malin et al. (2009), Hibberd et al. (2016), and Lynn et al. (2003) represent similar populations of low-threshold, wide dynamic range afferents that have intraganglionic laminar endings (Lynn et al., 2003).

Generally slower distension-evoked firing rates were associated with TRPV1-immunoreactivity and CGRPα expression (Malin et al., 2009). However, CGRPα occurred in a group of distension-sensitive, low-threshold colorectal afferents, similar to high frequency afferents (Hibberd et al., 2016). This group of afferents had wide dynamic firing ranges, but more modest distension-response profiles. Thus, they were described as “moderate frequency” afferents (Figures 3, 4). A proportion of capsaicin-sensitive, TRPV1-immunoreactive afferents (Malin et al., 2009) had threshold close to, or below 10 cm H_2_O, raising the possibility these cells represent a similar population to those described as moderate frequency (Malin et al., 2009). Similar to the high frequency afferents, moderate frequency afferents fit either the muscular and/or muscular/mucosal functional classifications. Indeed, subsets of lumbo-sacral low-threshold distension-sensitive afferents (muscular, and muscular/mucosal) have been identified as capsaicin-sensitive (Brierley et al., 2005a; Jones et al., 2005; Spencer et al., 2008a; Zagorodnyuk et al., 2011; Kiyatkin et al., 2013).

Malin et al. (2009) reported a group of distension-sensitive colorectal afferents that had relatively higher thresholds (~17.5 cmH_2_O/12.9 mmHg) (Malin et al., 2009), flatter distension-response profiles and slower firing frequencies. This group, referred to as “low frequency” afferents, were predominantly TRPV1-immunoreactive and capsaicin-sensitive. Colorectal afferents referred to as “low frequency” were also recorded by Hibberd et al. (2016) (Figures 3, 4). They had similarly flat distension response profiles, slower average firing rates, and expressed CGRPα. However, low frequency afferents recorded by Hibberd et al. (2016) had lower distension-response thresholds and tended to fire in bursts (Figure 3; Hibberd et al., 2016). The serosal functional class of afferents typically show relatively slow firing rates, flat distension-response curves and capsaicin sensitivity. Low frequency afferents could represent the serosal functional class. However, serosal afferent thresholds have been described as significantly higher than those tested by Malin et al. (2009) or Hibberd et al. (2016): ~45 mmHg in tube preparations (Hughes et al., 2009b), or the equivalent of 68–136 cm H_2_O in flat sheet preparations (Zagorodnyuk et al., 2011).

## Non-Responsive Afferents

Populations of afferents were responsive to pelvic or rectal nerve stimulation but not to distension or focal tissue compression. These cells remain to be characterized in detail and may include high-threshold serosal afferents, mechanically-insensitive silent afferents, and/or afferents whose axons pass through pelvic or rectal nerves en passant to other pelvic visceral organs. All such cells recorded by Hibberd et al. (2016) contained CGRPα and had electrophysiological properties otherwise similar to distension-sensitive colorectal afferents.

## Summary

The colon and rectum are innervated by a surprisingly diverse and distinct set of sensory afferent subclasses. These afferents innervate all layers of the colon and rectum and have activation thresholds ranging from the imperceptible through to the noxious range. Although further work is required to conclusively document the combined structure, function, and molecular profile of each afferent subtype within the splanchnic and pelvic nerves, our current knowledge provides us with fundamental information of how mechanical and chemical stimuli are detected within the colon and rectum. New technologies may also allow greater understanding of the functional properties of colonic afferents as well as the integration of their sensory inputs into higher levels (Makadia et al., 2018; Spencer et al., 2018). Future studies identifying how the structure, function, and molecular profile of each afferent subtype potentially changes in disease states will be important in the treatment of gastrointestinal disorders affecting the colon and rectum.

## Author Contributions

All authors listed have made a substantial, direct and intellectual contribution to the work, and approved it for publication.

### Conflict of Interest Statement

The authors declare that the research was conducted in the absence of any commercial or financial relationships that could be construed as a potential conflict of interest.
